# Where there is no evidence: use of expert consensus methods to fill the evidence gap in low-income countries and cultural minorities

**DOI:** 10.1186/1752-4458-4-33

**Published:** 2010-12-21

**Authors:** Harry Minas, Anthony F Jorm

**Affiliations:** 1 Centre for International Mental Health, Melbourne School of Population Health, University of Melbourne, Parkville, Victoria 3010, Australia; 2 Orygen Youth Health Research Centre, Centre for Youth Mental Health, University of Melbourne, Locked Bag 10, Parkville, Victoria 3052, Australia

## Abstract

**Background:**

In both developing countries and in relation to cultural minorities there have been calls to scale up mental health services and for evidence-informed policy and practice.

**Evidence based medicine:**

The evidence based medicine movement has had a major influence in improving practice. However, implementation of this approach has some major difficulties. One that has been neglected is the situation where there is no relevant evidence. This situation is more likely to occur for healthcare decisions in developing countries or for cultural minorities within developed countries, because resources do not exist for expensive research studies.

**Consensus methods:**

Consensus methods, such as the Delphi process, can be useful in providing an evidence base in situations where there is insufficient evidence. They provide a way of systematically tapping the expertise of people working in the area and give evidence that is readily applicable for a particular country and culture. Although consensus methods are often thought of as low in the hierarchy of evidence, consensus is central to the scientific process. We present four examples where the Delphi method was used to assess expert consensus in situations where no other evidence existed: estimating the prevalence of dementia in developing countries, developing mental health first aid guidelines in Asian countries, mental health first aid guidelines for Australian Aboriginal people, and modification of the concept of 'recovery' for Australian immigrant communities.

**Conclusion:**

Consensus methods can provide a basis for decision-making and considered action when there is no evidence or when there are doubts about the applicability of evidence that has been generated from other populations or health system settings.

## Background

Mental disorders are common in all countries, with considerable variation in reported prevalence across countries, even when the same methods for estimation of prevalence are used [1]. The treatment gap is wide everywhere, widest in developing countries [2,3]. Among the reasons for failure of coverage is massive under-investment in mental health service provision [4], resulting in serious shortages of health facilities and skilled mental health professionals [5], reliance on outmoded mental hospitals and lack of community mental health services [6], and frequent neglect and abuse of the human rights of people with mental illness [7-9]. This developing country profile of population mental health and mental health services - variability in prevalence, under-investment in provision of appropriate and accessible mental health services, low rates of utilisation of mental health services, and failures of rights protection - is replicated in the case of cultural minorities, even in wealthy countries with relatively well-developed mental health systems [10,11]. In both cases (developing countries and cultural minorities), there are legitimate concerns about the validity of 'western' diagnostic and treatment practices [12], and the applicability of 'western' models of mental health service [13]. Also in both cases there have been calls for scaling up mental health services [11,14] for new ways of working [15], and for evidence-informed policies and practice [14,16].

## Evidence based practice

Over the past 30 years, the evidence based medicine movement has had a major influence in improving practice. Evidence based medicine involves "integrating clinical expertise with the best available clinical evidence derived from systematic research" [17]. This movement has led to improvements in the reporting and registration of clinical trials, the provision of systematic reviews of controlled trials (e.g. the Cochrane Collaboration) and the development and promotion of clinical practice guidelines. While the movement began in developed countries, there has also been advocacy for evidence based medicine in developing countries [18]. It has been argued that the need for evidence based practice is vital in these countries because financial resources are scarce and need to be channelled to the best possible care options [19]. In particular there is a need for evidence-informed decision making by policy makers, health system managers and others who responsible for shaping health systems and delivering services [20].

Despite its many successes, the evidence based medicine movement has encountered some significant barriers in changing clinical practice, including the difficulty that practitioners have in finding and interpreting the evidence, and in applying it in practice, due to organisational barriers, patient adherence and lack of quality practitioner education [21]. There has also been criticism of the movement because of its emphasis on high quality evidence from randomised controlled trials, which tend to be carried out in ideal circumstances that may be remote from everyday practice. This has led to calls for more "practice-based evidence", where the evidence is gathered in real-life clinical settings and there is greater emphasis on the external validity of the evidence (generalisability) rather than on its internal validity (validity of causal inference) [22]. An additional issue is the relative (compared to biomedical research) scarcity of health system research and evidence for policy and health system design [23-25].

Here we discuss an obvious, but neglected, barrier to evidence based practice--situations where there is no evidence [26]. This barrier can be found for many areas of healthcare, but is more likely to be found in developing countries and for cultural minorities within developed countries.

Developing countries have limited resources to support research [27]. Therefore, evidence is often imported from developed countries, raising the issue of applicability of this evidence in settings and from populations other than those in which it was generated. Where the evidence reflects universal biological processes (e.g. infection and immunisation), it will be possible to generalise, but where it involves social and cultural processes (e.g. persuading people to control infection or to immunise), it may not be. In psychiatry, where social and cultural factors are integral in making healthcare decisions and developing systems of care, much of the evidence from developed countries will be limited in generalisability. The same applies to cultural minorities within developed countries. Although it may be possible to develop an adequate evidence base for the mainstream population, the resources may not exist to do this for small indigenous or immigrant groups. In addition, immigrant and refugee communities that do not speak the host country language are frequently excluded from research because of the difficulties and expense of translation.

## Expert consensus

The development of a locally relevant evidence base using expert consensus is a valuable approach where other evidence is unavailable. While developing countries may not have the resources to carry out randomised controlled trials, population surveys, cohort studies or health service evaluations [27], they do have considerable local experience derived from practice. The same is true for cultural minorities in developed countries. There are formal methods of developing consensus from experts, such as the Delphi process, nominal group technique [28] and consensus conference methods [29], which can be used to harness this experience. These methods have acceptable construct validity [30] and reliability [31]. By using these methods to develop an evidence base that can guide decisions, policy makers and practitioners can move beyond relying on their own experience and drawing on the accumulated experience of a larger, expert group.

As well as being feasible with limited resources, consensus methods overcome some of the limitations that have been identified with evidence based medicine. They embody the principle of practice-based evidence, provide results that are relevant to the local population and culture, and are readily implementable within the healthcare system. This approach fits with the call to improve practice by capitalising on accumulated practical experience and using this to develop better interventions.

The Delphi consensus method has been widely used in disparate fields to inform: policy-making [32,33]; design of health services [34,35]; development of diagnostic guidelines and protocols [36,37]; developing mental health first aid guidelines for psychosis [38,39], suicide [40-42], self-injury [42], and panic attacks [43]; and service and research priority-setting [44,45]. In relation to dementia, the Delphi consensus method has been used to clarify issues and identify consensus in diagnosis and clinical assessment [46], treatment and management [47-49], services, outcomes [48], research, and to estimate prevalence [50] in settings where the necessary epidemiological studies have not been done.

There are several possible objections to the adequacy of expert consensus as a source of evidence. The evidence based medicine movement has been associated with a hierarchy of levels of evidence. Typically, the strongest form of evidence is held to be a meta-analysis of randomised controlled trials, with expert consensus ranked at the bottom of the hierarchy. However, while systematic reviews and meta-analyses are held to be the highest form of evidence, there is little agreement on the reporting of a critically important component of the systematic review process - the search methods used in carrying out systematic reviews [51]. Even where relevant systematic reviews exist, there are often questions about the applicability of the findings of the systematic review to the task of choosing among policy or program options in particular circumstances [52].

If expert consensus is so weak, should we be proposing it as a strategy for contributing to informed decision-making? An important counter-question to ask is: how were some types of evidence assigned to be higher in the hierarchy than others? The answer is "expert consensus". Expert consensus is seen as adequate to validate hierarchies of evidence or indeed other components of the evidence based medicine enterprise, such as the CONSORT Statement for reporting randomised controlled trials [53] and principles for developing practice guidelines [54].

More generally, consensus has an important role in the scientific process. New theories gain ground as more members of the scientific community see a new theory as giving a better account of the evidence than older ones [55]. The role of consensus can be seen in the past rise of scientific developments like continental drift and prions as infectious agents, but also in contemporary challenges such as climate change [56].

A paradox in recommending consensus in order to make progress is that consensus may hinder progress and promote no longer useful or even harmful practices. Consensus is an expression of the prevailing political, social, cultural, scientific world view of the time. There may, for example, be a broad consensus within society with which scientists do not agree, or there may be a consensus among experts that produces undesirable outcomes [57]. The validity or probity of consensus-based decisions can be called into question, as has famously been done recently in relation to the work of the Inter-Governmental Panel on Climate Change (IPCC) which, as a global body of 800 climate scientists, relies on reaching consensus [58].

The issue is not whether consensus *per se *is weak or strong, because it is integral to how science works, but rather what foundation of evidence the consensus rests on. A consensus based on a set of randomised controlled trials [50] may well be better than a consensus based on personal clinical experiences. What we are proposing here is not that expert consensus is as good a source of evidence as a meta-analysis of trials, but that it is a better source of evidence than the experience of a single individual, which is the alternative when there is no other evidence available. Expert consensus may well turn out to be wrong. Indeed, the history of science indicates that it inevitably will. However, it presents a better basis for guiding action, because group consensus will generally produce better judgements than any individual's judgement [59]. Expert consensus methods are a step on the road to informed decision-making. When better forms of evidence become available, these should of course be relied upon.

## Examples of how expert consensus can be used

To illustrate the use of expert consensus methods in situations where there is no evidence, we describe four projects that have used the Delphi process to fill evidence gaps in developing countries and with cultural minorities.

### Estimating the prevalence of dementia

Studies of the prevalence of dementia are major research undertakings. Estimates of prevalence rates are available from well-conducted studies in most developed countries, but for the rest of the world there is limited or no evidence. To overcome this lack, Alzheimer's Disease International carried out a Delphi study with a panel of 12 international experts [50]. The panel members were provided with a systematic review of the published studies and, on the basis of those studies and additional information of possible relevance such as development status and child and adult mortality in the regions, were asked to estimate prevalence for every WHO world region for 5-year age groups from age 60 onwards. "When published information is scarce, experts can make inferences using other data from comparable contexts" [50]. The consensus estimates of prevalence led to an estimate that 24 million people had dementia in 2001 and that 60% of them lived in developing countries, with this rising to 71% by 2040. These prevalence estimates are currently the best available basis for making policy and planning services. In particular, they raise awareness of the need for planning in relation to the rapid rise in number of dementia cases in developing countries. The purpose of the study was to generate estimates that are "the best currently available basis for policymaking, planning, and allocation of health and welfare resources" [49].

### Mental health first aid in developing countries

Mental health first aid is the help provided to a person who is developing a mental health problem or is in a mental health crisis. A Mental Health First Aid training course has been developed in Australia and has spread to many other countries [42]. Mental health first aid guidelines have been developed for English-speaking countries and used as a basis for the Mental Health First Aid training curriculum [60], but these may not be generalisable to countries with very different cultures and health systems. As a low-cost solution to this problem, psychosis first aid guidelines were developed using the Delphi method with clinicians from a wide range of Asian countries [39]. Because guidelines need to be specific to particular countries, more recent work has used the Delphi process to develop suicide first aid guidelines for India [40], the Philippines and Japan, using the consensus of panels of clinicians from each of these countries.

### Mental health first aid with Aboriginal Australians

A related example concerns Aboriginal Australians, who comprise less than 3% of the Australian population, but are a priority for action because they are disadvantaged in both physical and mental health. A Mental Health First Aid training program has been developed for Aboriginal and Torres Strait Islander peoples and found to be culturally acceptable [61]. However, the first aid actions recommended in the training were based on a modification of those for the mainstream Australian community. To develop more culturally appropriate mental health first aid, a Delphi study was carried out with an expert panel of Aboriginal mental health professionals [61]. Panel members were presented with statements about possible first aid actions and were encouraged to suggest other actions not already covered by the survey content. The endorsed statements, covering depression, psychosis, suicidal thoughts and behaviours, deliberate non-suicidal self-injury, trauma and loss, and cultural considerations in giving mental health first aid, were used to write guidelines and a revised curriculum for Aboriginal and Torres Strait Islander Mental Health First Aid training [62].

### The construct of 'recovery' in immigrant communities

Mental health policy, particularly as it relates to the mental health NGO sector that provides rehabilitation, social support, housing, employment and related services, increasingly requires that recovery principles should underpin service design and delivery. However, the origins of the recovery movement are in western Anglophone countries, and recovery principles reflect the cultural commitment of these countries to values such as privacy and confidentiality, individual autonomy, and self-determination. The relevance of these underlying values to many immigrant communities in Australia, which tend to place greater value on interdependence, collective decision-making, and authority of the senior member of the family, has been called into question, therefore challenging whether the current recovery construct can be applied in a multicultural society. This study [63] used the Delphi method, with senior staff of a major NGO constituting the expert panel, to explore ways in which the construct of recovery may need to be modified to make it more relevant for a culturally diverse population of clients. The study also focused on ways in which a modified conception of recovery can inform the design and delivery of mental health services by the organisation. It is an example of use of the consensus method to inform organisational change for service improvement.

## Conclusion

Evidence is most important as a basis for decision-making and action, such as medical treatment of an individual, decisions about health policy, or actions such as investment in one rather than another form of service delivery. It is clear that such decisions will be made and actions embarked upon whether or not there is evidence or whether the decision-maker or practitioner is familiar with such evidence as does exist. Consensus methods can provide a basis for decision-making and considered action when there is no evidence or when there are doubts about the applicability in a particular setting of evidence that has been generated from other populations or health systems. The issue is not whether consensus methods provide evidence that is as good as other ways of generating evidence, but whether the evidence generated by using such methods is better than no evidence or inapplicable evidence.

Beyond questions of the availability or quality of evidence, expert consensus methods have a particular advantage when applied in fields that are neglected and that require urgent action, such as mental health system development in low-income countries and for immigrant and refugee communities. The process of identifying consensus shifts the focus away from areas of disagreement, which frequently are many, to areas of agreement. If key stakeholders, such as government decision makers, service users, as well as clinicians and researchers, are involved in the expert panel considering a particular issue, this can facilitate informed decision-making and action.

## Competing interests

The authors declare that they have no competing interests.

## Authors' contributions

HM and AJF contributed equally to the conception and writing of this paper. Both authors read and approved the final manuscript.
